# Endogenous Labelling of Extracellular Vesicles and Image Capture of Their Interactions With Acceptor Cells

**DOI:** 10.1002/cbic.70292

**Published:** 2026-04-04

**Authors:** Eden Booth, Massimiliano Garre, Dan Wu, Donal F. O’Shea

**Affiliations:** ^1^ Department of Chemistry RCSI University of Medicine and Health Sciences Dublin Ireland

**Keywords:** BF_2‐_azadipyrromethene fluorophore, cell uptake, confocal and lifetime microscopy, endogenous labelling, extracellular vesicles

## Abstract

Despite their significance in both physiological and pathological contexts, the mechanisms governing extracellular vesicle (EV) uptake and cytosolic cargo delivery remain incompletely understood. Here, we report the development of a BF_2_‐azadipyrromethene‐based near‐infrared fluorophore for the endogenous labelling of EVs, enabling their interaction with acceptor cells to be observed using both intensity and fluorescence lifetime imaging microscopies. Through endogenous labelling, dye aggregate‐free EVs can be readily isolated and confirmed through their emission wavelengths (λ_max_ 720 nm) and lifetime (2.7 ns). These photophysical properties permitted clear discrimination between aggregated and disaggregated forms of the fluorophore in complex biological environments. Endogenously labelled EVs containing the disaggregated fluorophore were incubated with unlabelled acceptor cells and imaged by both intensity and lifetime microscopy, confirming identical intensity and lifetime values to the free disaggregated dye. Incubation at 4°C, which slows cellular processes, enabled visualisation of initial EV–plasma membrane interactions, while at 37°C efficient transfer of the fluorophore into acceptor cells was observed, evidenced by increased emission intensity and matching characterised spectral–lifetime signatures. Our results establish endogenous labelling as an efficient method to introduce a fluorophore into the lumen of EVs, thereby providing a robust platform for real‐time visualisation of EV communication with acceptor cells.

## Graphical abstract

Isolation and characterisation of endogenously labelled extracellular vesicles (EVs) from donor cells are timelapse imaged using intensity and lifetime microscopies to capture EV contact with filopodia and the plasma membrane (PM) of acceptor cells. Accumulation of EVs within and around filipodia could be observed and following passage through the PM accumulation within trafficking vesicles close to the inner leaf was recorded.

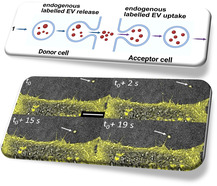

## 1 Introduction

Extracellular vesicles (EVs) are lipid‐enclosed compartments which are secreted by cells into the surrounding extracellular space to facilitate cell‐to‐cell communication [1, 2, 3]. EVs represent a heterogeneous group of vesicles, comprising diverse subpopulations that vary in size, composition and molecular cargo [4, 5, 6]. This heterogeneity, along with the phenotypic diversity of EVs, remains poorly understood, which underscores the importance of further investigation [7, 8, 9, 10, 11]. Recent studies have increasingly focused on the importance of both the smaller exosomes (30–100 nm) originating from the endosomal compartment and the larger micro‐vesicles (100–400 nm) generated by budding from the plasma membrane (PM), due to their involvement in a wide array of biological processes, as well as their potential as biomarkers for various diseases [5, 12, 13]. It is of profound scientific interest and medical importance that EVs can transfer biomaterials from donor to acceptor cells, with the potential of causing a phenotypic change in the acceptor cells [14, 15, 16]. Their potential for use in therapeutics and diagnostics was highlighted, with their ability to transfer oncogenic material between cells documented [17, 18, 19, 20, 21]. Over the years, EVs have increasingly garnered attention for their capacity to modulate immune responses, establishing them as critical components in therapeutic interventions relating to autoimmune diseases and cancer immunotherapy [15, 22, 23, 24]. Owing to the different physiological and pathological states of donor cells, the complex variations between their compositions, functional properties and sizes have posed a series of obstacles in the effort to standardise techniques for functional analysis of EVs [25, 26]. Furthermore, the capability to trace EV trajectory in vitro and in vivo has been restricted due to technical limitations with current imaging techniques, including the lack of effective methods to preserve EV structural and chemical integrity during their labelling protocols [27, 28, 29]. For example, label introduction through binding recognition or covalent linkages to EV surface proteins causes chemical modification of these proteins, which can interfere with experimental measurements [30].

**FIGURE 1:**
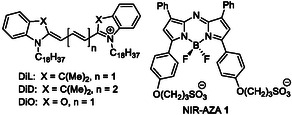
Structures of dialkylcarbocyanine dyes DiL, DiD and DiO used for passive exogeneous labelling of EVs and NIR‐AZA **1** developed for active endogenous labelling of EVs.

Due to its excellent spatiotemporal resolution, live cell fluorescence microscopy with molecular labelling offers a prominent means of studying EVs and their interactions in living organisms and cells [31]. As with most imaging platforms, there exist several hurdles when utilising fluorescence‐based methods for in‐depth EV investigations such as background fluorescence and lack of labelling specificity [32, 33]. The most widely used approach for molecular fluorophore labelling of EVs is via exogenous passive treatment of purified EVs with dialkylcarbocyanine lipophilic membrane dyes such as DiL, DiD and DiO, or the structurally similar PKH26 and PKH67 (Figure 1) [34, 35, 36].

**FIGURE 2:**
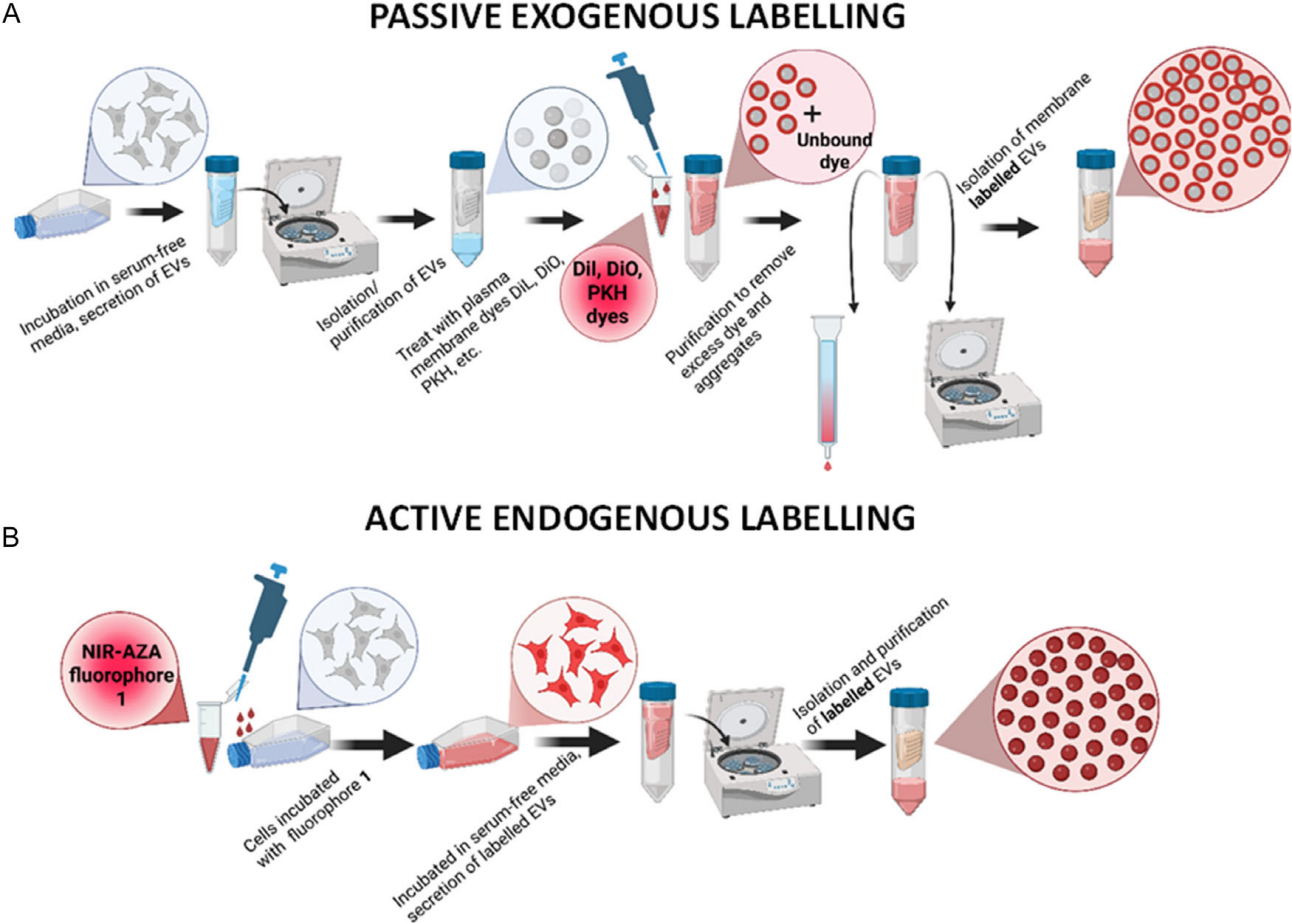
Comparative schematic of (A) passive exogeneous and (B) active endogenous EV labelling with molecular fluorophores.

Such labelling is carried out by directly treating isolated EVs with a large excess of the dye of choice which necessitates further purification to remove excess dye through centrifugation or chromatography (Figure 2A) [37]. Several limitations of this passive exogenous approach are recognised, as purifications are not always successful which can result in sample contamination with dye aggregates and possible alteration of the vesicles’ surface functional properties [38, 39, 40, 41, 42]. In particular, dye aggregates, which form spontaneously in aqueous solutions, accumulate on EV membranes and can have similar sizes to EVs. This has been shown to give rise to false‐positive fluorescent signals, making experimental data interpretation a challenging task [34, 43]. We previously communicated a novel endogenous EV labelling strategy, in which donor cells autonomously internalise and incorporate a fluorophore into EVs prior to their secretion, offering a more physiologically relevant and artefact‐free alternative to conventional passive exogenous labelling techniques (Figure 2B) [44]. This endogenous labelling approach employs the BF_2_ azadipyrromethene fluorophore, NIR‐AZA **1**, as an amphiphilic highly photostable near infrared (NIR) fluorophore with emissions beyond 700 nm [45, 46, 47]. The structure of **1** possesses two sulphonic acid groups tethered via an aliphatic spacer, conferring a distinct amphiphilic character: a hydrophilic, negatively charged sulphonate‐rich domain and a hydrophobic region decorated with phenyl substituents (Figure 1). The negatively charged sulphonic acids facilitate incorporation into cell membranes by ionic association with the ammonium component of the zwitterionic membrane phospholipids and within the aqueous compartments of vesicles, thereby allowing for integration into EVs. In use, NIR‐AZA **1** is first incubated with EV donor cells until they reach saturation, then cell media is removed, and the labelled cells are washed to remove unincorporated dye and then incubated in serum‐free media for 48 hr allowing EV biogenesis to occur. Labelled EVs are then harvested and purified with label efficiencies of 88% reported, underscoring the robustness of this approach [44]. Importantly, because excess fluorophore is eliminated prior to EV collection, this avoids dye aggregation and false‐positive artefacts, a known limitation of exogenous dyes like PKH26 and DiI, which can co‐isolate with EVs even after purification [48].

**FIGURE 3:**
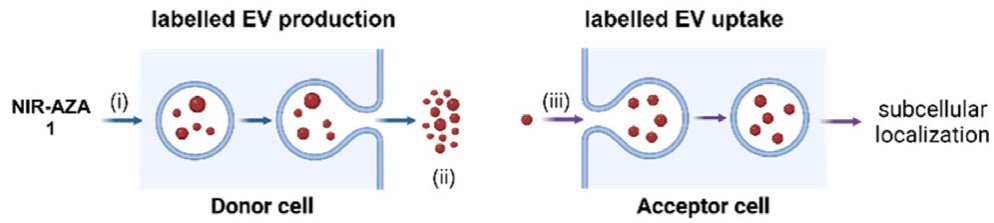
Schematic for confocal and FLIM imaging experiments of (i) endogenous labelling and (ii) characterisation of EVs from donor cells, followed by (iii) their uptake and distribution within acceptor cells.

Confocal laser scanning microscopy (CLSM) imaging is particularly valuable for the study of EVs, enabling precise localisation and dynamic interaction analysis [49, 50]. In parallel, fluorescence lifetime imaging microscopy (FLIM) offers a complementary approach for studying EVs in cellular environments, as it enables differentiation of fluorescence signals based on lifetime rather than intensity which is sensitive to the fluorophore microenvironment [51, 52]. In this work, fluorescence emission and lifetime spectroscopy measurements are used to determine identifiers of the monodisperse and aggregated forms of NIR‐AZA **1** for use in interpreting microscopy imaging data from live cell and EV experiments. Together, intensity and lifetime microscopy techniques were employed for dynamic imaging studies of (i) the uptake of **1** into donor cells, (ii) the isolated purified endogenously labelled EVs obtained and (iii) the first interactions and uptake of EVs into acceptor cells (Figure 3). It was envisaged that the results obtained from using both imaging modes with endogenously loaded EVs could introduce novel perspectives for decoding the complex relationships of EV–cell interactions. This could help develop further translational uses for EVs.

## 2 Results and Discussion

### 2.1 Solution Phase Characteristics of NIR‐AZA 1

**FIGURE 4:**
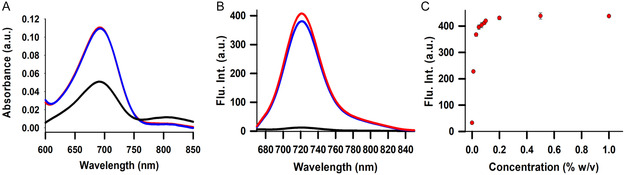
Solution absorption and emission characteristics of **1** at 5 µM concentration. (A) Absorption spectra of **1** in MeOH (red), DMEM (black) DMEM plus 0.1% PS‐20 (blue). (B) Emission spectra of **1** in MeOH (red), DMEM (black) and 0.1% PS‐20 (blue). (C) Plot of fluorescence emission intensities of **1** in DMEM with increasing concentrations of PS‐20 (*n* = 5). Error bars represent ±1 SD.

To investigate the spectroscopic characteristics of **1** in aqueous extracellular medium and a protic organic solvent, 5 µM solutions of the fluorophore were prepared in Dulbecco's Modified Eagle Medium (DMEM) and MeOH. In MeOH, **1** exhibited an absorption maximum at 683 nm with a corresponding emission maximum at 720 nm (Figure 4A,B). Divergent from these results, the solution of **1** in DMEM displayed relatively broader absorption and a 32‐fold weaker emission, with maxima at 686 nm and 720 nm, respectively (Figure 4A,B). Additionally, when excited at the longer wavelength of 780 nm, a second emission centred at 823 nm was observed (Figure S1). Further investigation of this long‐wavelength emission showed that it was also observable in phosphate‐buffered saline (PBS), centred at 821 nm, demonstrating that aggregation is intrinsic to the dye in aqueous media and does not require biomolecular constituents (Figure S1). The mechanism of J‐aggregation is promoted through the amphiphilic nature of **1** containing a sulphonic acid substituted hydrophilic face and a phenyl substituted hydrophobic face (Figure S1). These results show that in MeOH, **1** is fully disassociated whereas in aqueous cell media a degree of self‐association occurs due to its amphipathic characteristics. Next, the behaviour of aqueous DMEM solutions of **1** with respect to the inclusion of neutral surfactant was explored using polysorbate 20 (PS‐20) which has a documented critical micelle concentration (CMC) of 0.06% (w/v) [53]. This analysis was conducted by performing a titration of PS‐20 into a DMEM solution of **1** while monitoring emission changes. The emission intensity at 720 nm increased sequentially up to 0.1% w/v of PS‐20 and then plateaued beyond that concentration (Figure 4C). Notably, the emission profile and intensity were almost identical to those measured in MeOH, showing that the aqueous environment influences the degree of aggregation of **1** and, as a result, its emission properties. This informative result illustrates that **1** would likely be strongly emissive in mixed lipid cellular environments.

**FIGURE 5:**
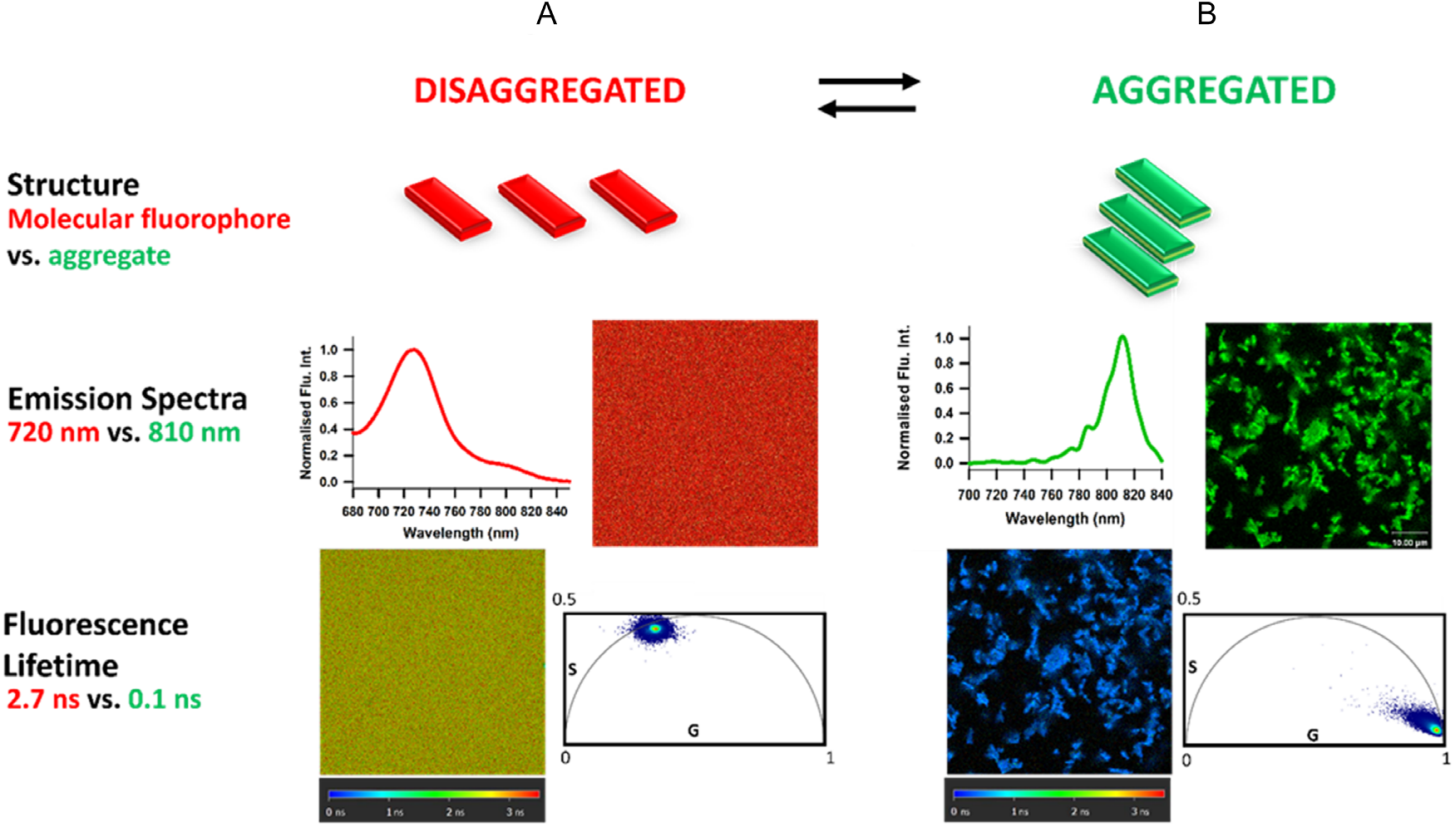
Microscopy characterisation of **1** (5 µM) in MeOH and DMEM. (A) In MeOH, schematic descriptor of disaggregated form with emission spectrum, intensity and FLIM images and phasor plot lifetime analysis. (B) In DMEM, schematic descriptor of aggregated form with emission spectrum of an aggregate (scale bar 10 µm), intensity and FLIM images and phasor plot lifetime analysis.

In anticipation of live cellular imaging, an assessment of the MeOH and DMEM solutions was conducted visually and spectroscopically using confocal and FLIM microscopies. Both solutions were subjected to laser excitation at 684 nm, and two spectrally distinct emission regions collected between 700–750 nm and 800–850 nm. An emission intensity between 700 and750 nm was obtained for the MeOH solution with a maximum at 720 nm as previously obtained on the fluorometer (Figure 5A). Conversely for the DMEM solution, an emission between 800 and 850 nm was revealed from a polydisperse collection of aggregates, with emission maximum at 810 nm (Figure 5B). As a result of its ability to self‐associate and disassociate depending on the hydrophilicity of the surrounding medium, fluorophore **1** is evidently dual emissive in nature. The phasor lifetimes of **1** in MeOH (disassociated form) as well as DMEM (aggregated form) were measured at 2.7 and 0.1 ns, respectively (Figure 5A,B). This ability to distinguish between aggregate and molecular fluorophore forms using FLIM was envisaged to be a valuable tool for more complex cellular and EV imaging experiments.

**FIGURE 6:**
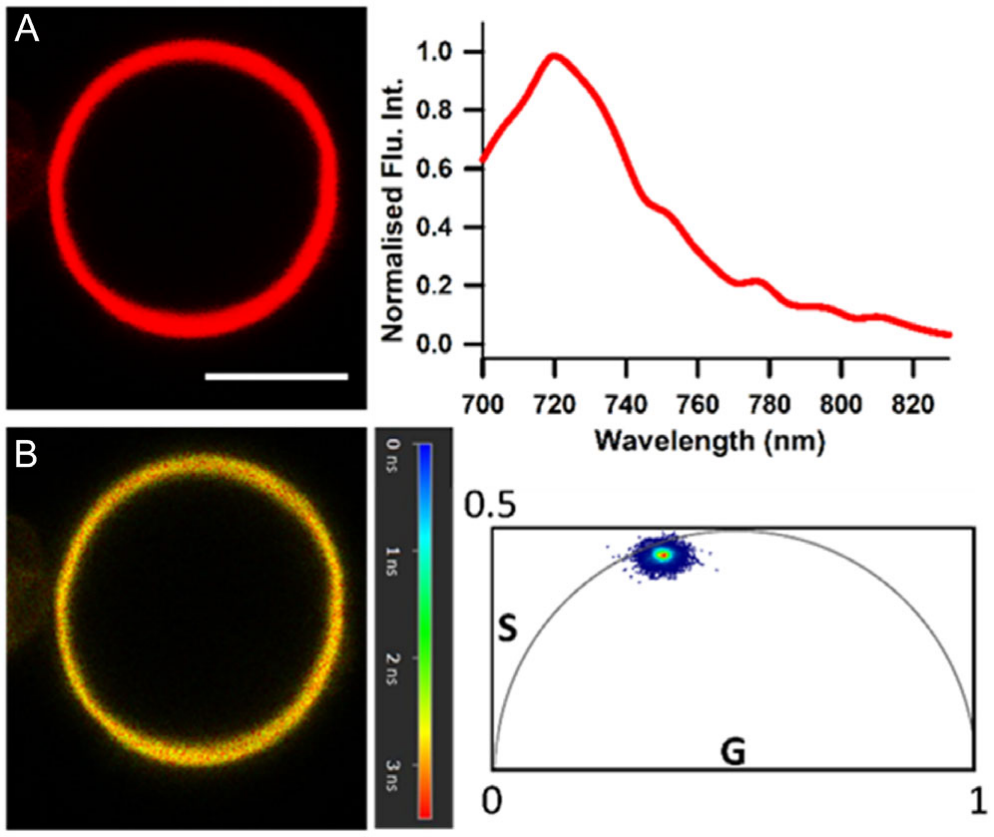
Photophysical characterisation of **1** in synthetic liposomes. (A) Fluorescence intensity image of liposome (scale bar 10 µm) with corresponding emission spectrum. (B) FLIM image of liposome shown in (a) with corresponding phasor plot indicating 2.7 ns lifetime.

The photophysical properties of **1** in synthetic liposomal vesicles was next studied. Liposome populations were produced from L‐α‐phosphatidylcholine, stearylamine and cholesterol (7:2:1) and treated with a PBS solution of **1** to a concentration of 5 µM. The labelled liposomes were immobilised onto poly‐lysine‐coated microscope slides, and emission spectra, intensity and lifetime images were recorded. Confocal images showed the liposome structure, with emission only observable from the bilayer and not the hydrophilic core. The emission spectrum had a maximum at 720 nm indicating the disassociated form preferentially existed within this mixed lipid environment (Figure 6A). This was further confirmed through measurement of a fluorescence lifetime of 2.7 ns which was comparable to the value obtained for a MeOH solution (Figure 6B). Hence, it can be inferred that **1** can embed itself into a lipid bilayer as its dissociated form, and this can be established via intensity and lifetime imaging data offering a means to study this in more complex scenarios.

### 2.2 Cellular Uptake of NIR‐AZA 1 into EV Donor Cell Line

For this investigation, the human triple‐negative breast cancer cell line MDA‐MB‐231 was selected as the EV donor, as their EVs have been shown to facilitate intercellular communication and influence tumour progression, metastasis and therapeutic responses [54, 55, 56, 57, 58, 59]. In addition to transferring microRNAs that can regulate acceptor cells and potentially modulate their behaviour, it has been discovered that EVs from these cells induce pro‐inflammatory features in tumour‐associated macrophages [60].

Conversely, MDA‐MB‐231‐derived EVs have also been observed to polarise RAW264.7 cells into M2 macrophages, inducing a shift towards an anti‐inflammatory state promoting lymph node metastasis [61]. Such context‐dependent and seemingly contradictory immunomodulatory outcomes underscore the complexity of EV‐mediated signalling and highlight the critical need to characterise these interactions in a cell‐type specific manner. As such, it was considered that a novel approach to spatial and temporal imaging of the interactions between endogenously labelled MDA‐MB‐231‐derived EVs with different acceptor cells would be of value. Two different acceptor cell lines, RAW 264.7 macrophages and HeLa Kyoto cells, plus the parental MDA‐MB‐231 cells themselves, were employed as acceptor cells allowing cross comparisons to be made.

**FIGURE 7:**
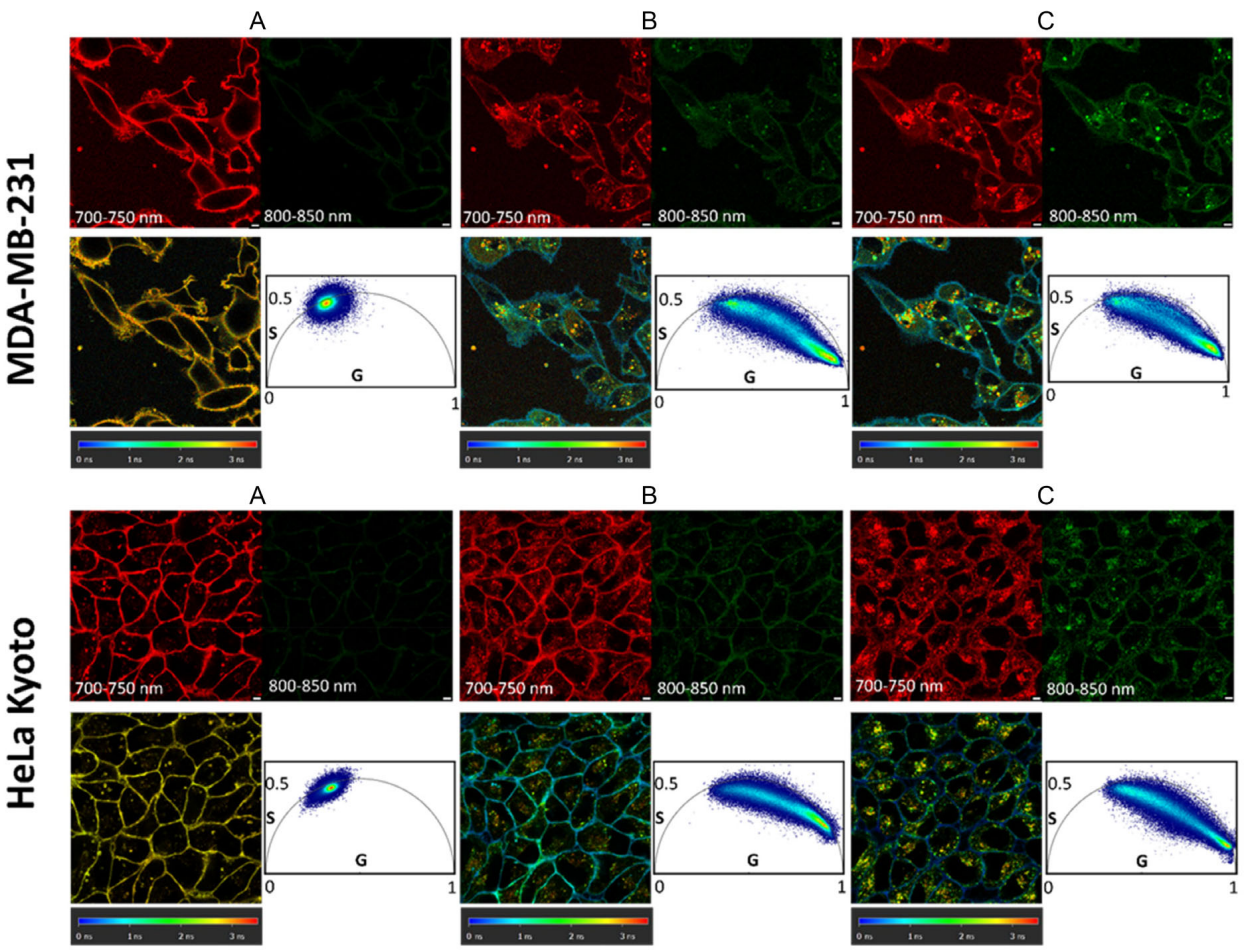
Time course of intensity and lifetime imaging of the uptake of **1** into EV doner MDA‐MB 231 cells (top) and HeLa Kyoto cells (bottom). Representative images acquired at (A) 5 min, (B) 30 min and (C) 2 h. Top row: steady‐state fluorescence intensity images acquired in two spectrally distinct regions – 700–750 nm (red channel) and 800–850 nm (green channel). Bottom row: corresponding FLIM images and phasor lifetimes at 5 min, 30 min and 2 h (left to right). (scale bar 5 µm).

Having clearly distinguished the fluorescence emission and lifetime characteristics of the disassociated and aggregated forms of **1**, this knowledge was utilised to interpret dynamic live cell imaging data commencing with its uptake and intracellular trafficking as the first step of our endogenous labelling. We have previously reported that a 5 μM concentration of **1** is optimal for greater than 88% EV labelling so, for this portion of the investigation, the donor MDA‐MB‐231 cells were treated with **1** at this concentration and imaged during its uptake and intracellular distribution [44]. To facilitate these real‐time live cell experiments, chamber slides seeded with cells were appropriately positioned on the microscope stage, which was fitted with an incubator to maintain the surrounding temperature at 37°C and CO_2_ at 5%. After selecting a suitable imaging field of view (FOV), the cells were treated with **1** in pre‐warmed DMEM, and time‐lapsed fluorescence images and lifetime measurements were acquired between 5 min and 2 h. It was observed that at 5 min, upon interacting with the PM as the first point of contact between **1** and the cell, a strong emission from 700–750 nm, with lifetime of 2.7 ns, was observed (Figure 7A). This could confidently be ascribed to the association of the monomeric form of **1** with the lipid bilayer, just as detected with the liposomal simulation (Figure 6). At the 30 min mark, the fluorophore was observable both in the PM and the cytoplasm, where it accumulated in intracellular organelles. The collected emission now showed two distinct bands in the 700–750 and 800–850 nm ranges with the phasor lifetime plot distributed from 2.7 to 0.1 ns (Figure 7B). This can be attributed to the accumulation of aggregated **1** from the media into the PM. At the 2 h time point, again both emissions are recorded, and the phasor plot cloud was further shifted to the lower lifetimes indicating an increasing amount of aggregated **1** within internal vesicles. These results were revealing with respect to reported uses of passive exogenous labelling of EVs in which the challenges posed by fluorophore aggregation for the interpretation of imaging results have been established [40, 41, 42, 62]. Importantly in the case of **1**, as the aggregates and molecular forms are distinguishable from each other, both within media and cells, it was anticipated that this would also be the case for isolated labelled EVs.

As can be seen sequentially with the passing of time, **1** internalises into the cells with a concurrent change in phasor lifetime from a focus at 2.7 ns to a distribution between 2.7 and 0.1 ns. Importantly, the FLIM data can reveal the more complex and dynamic profile of emission, allowing both forms of the fluorophore to be temporally mapped to cellular regions. This evidence demonstrates that over time there is a gradual accumulation of aggregates in the PM and within some intracellular organelles. This ability to identify both forms of **1** was confirmed in a second (non‐EV donor) cell line (HeLa Kyoto), and the same temporal imaging profile for both intensity and phasor lifetimes was observed (Figure 7).

### 2.3 Isolation of Endogenously Labelled EVs

**FIGURE 8:**
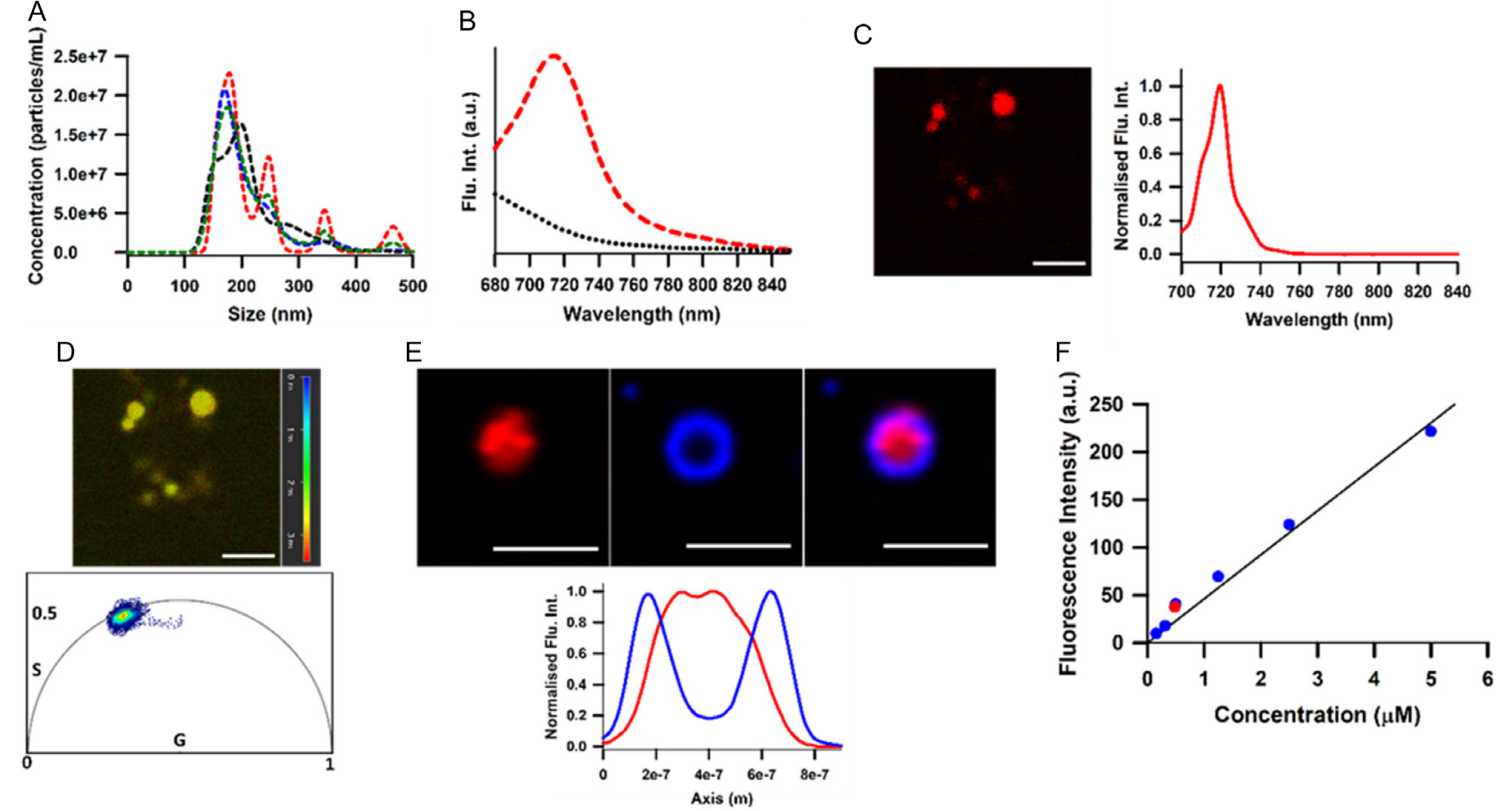
Spectroscopic characterisation of isolated labelled EVs. (A) Representative concentrations and NTA size distribution of isolated EVs. Red, green, blue and black dashed curves represent independent nanoparticle tracking analysis runs, demonstrating run‐to‐run reproducibility of the size distribution. (B) Spectrometer recorded fluorescence spectra of isolated EVs (red) and filtrate post‐isolation (black). (C) Fluorescence intensity image of EVs with corresponding emission spectrum (scale bar 1 µm). (D) FLIM image and corresponding phasor plot of EVs. (E) Co‐staining images of EVs with anti‐CD63‐AlexaFluor‐488 (blue) and endogenously labelled EVs with **1** (red), overlay (right). (scale bar 1 µm) Line intensity profile illustrating the localisation of AlexaFluor488 (blue) and fluorophore 1 (red). (F) Standard plot (blue circles) of varying concentrations of **1** in DMEM, fluorescence intensity of EVs in serum‐free media at 0.5 µM (red circle).

Following a 2 hr incubation, complete DMEM media was removed, and the MDA‐MB‐231 cells were washed three times with pre‐warmed PBS before adding serum‐free media, thus removing all extracellular **1**. Incubation was then continued for a further 48 h, allowing the biogenesis and secretion of EVs from donor cells. Following EV efflux, microscopy images of the donor cells showed that a small amount of **1** remained localised within larger intracellular organelles mostly in its disaggregated form (Figure S2). Fluorescent EVs were isolated from the conditioned medium by centrifugation employing 100 kDa molecular weight cut‐off centrifugal filters. This approach was chosen to avoid variability in yield often associated with ultracentrifugation [27, 63, 64]. Following this efficient protocol, the batch‐to‐batch concentration of isolated labelled EVs ranged between 2.25 × x10^7^ vesicles/mL and 0.25 × 10^7^ vesicles/mL (Figure 8A). As expected, EV sizing using nanoparticle tracking analysis (NTA) gave the largest distribution centred at 190 nm with smaller populations at 252, 356 and 470 nm. Spectrometer recorded emissions of the EVs displayed a single emission band with maximum at 720 nm, consistent with the data obtained for the fully dissociated form of **1** (Figure 8B). Following EV immobilisation on microscope slides, a single emission band centred at 720 nm and phasor lifetime of 2.7 ns were recorded. This confirmed that **1** existed in its monomeric form within EVs and that they were free of fluorophore aggregates (Figure 8C,D). The presence of tetraspanin CD63 on EV membranes was confirmed by their treatment with AlexaFluor‐488 labelled anti‐CD63 following which dual wavelength images were acquired [65, 66]. Upon exciting the EVs at 490 nm, an antibody emission centred at 525 nm was observable, which imaged as a ring localised around the outer surface of the EVs (Figure 8E, blue image). Comparison of the spatial positioning of both emissions through their line intensity profiles revealed a partial overlap between the AlexaFluor‐488 (blue trace) and **1** (red trace) emissions, suggesting that **1** is localised within the EV interior (Figure 8E). Prior to use in cell experiments, the concentration of **1** within EV samples used for cell imaging was standardised to be 0.5 µM, which was ten‐fold lower than that used in the labelling protocol, through comparison with a concentration versus fluorescence intensity plot of **1** (Figure 8F).

### 2.4 Treatment of Acceptor Cells with Endogenously Labelled EVs

With endogenously labelled EVs in hand, time course studies of their interaction with three different acceptor cell types were undertaken. Our goal was to track initial contact with the PM, uptake, cytosolic accumulation and the fate of the fluorescent cargo over time. For comparison, imaging experiments were also conducted using **1** alone, at the same 0.5 μM concentration as that contained within the EV solutions. By comparing fluorescent cargo inside and outside EVs at the same concentration, it could be determined whether their image profiles differed from each other.

**FIGURE 9:**
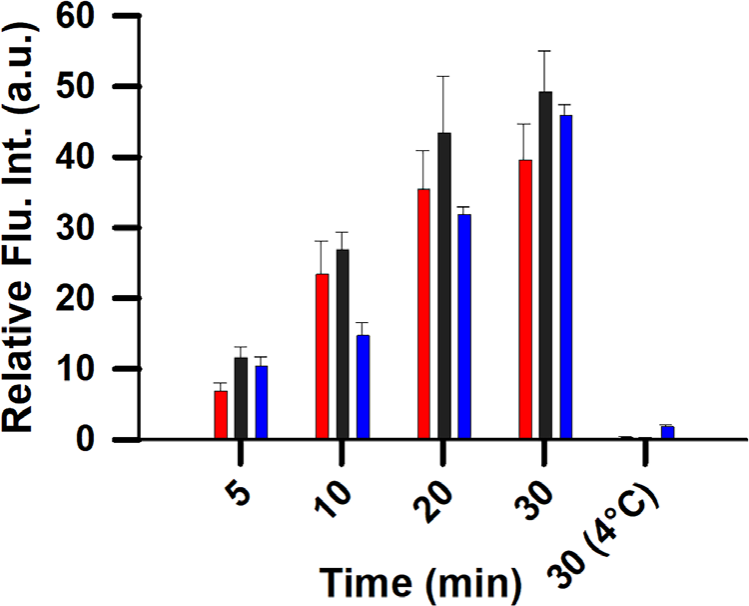
Plot of the fluorescence intensity of acceptor cells between 0and 30 min following treatment with endogenously labelled EVs from MDA‐MB 231 cells. RAW 264.7 cells (red bars); HeLa Kyoto cells (blue bars); MDA‐MB‐231 cells (black bars) at 37°C and 4°C. Error bars represent ±1 SD.

First, the progressive increase in acceptor cell fluorescence intensity was assessed from the first introduction of EVs up to 30 min. Plots of the fluorescence intensity from images acquired at 5, 10, 20 and 30 min revealed a steady increase over time, confirming the effective uptake of the EV delivered **1** into all three acceptor cell types (Figure 9). When uptake experiments were conducted at 4°C, conditions known to inhibit energy‐dependent cell uptake processes such as endocytosis, acceptor cell fluorescence was minimal at 30 min (Figure 9, 4°C data). This confirmed that, as expected, EV uptake was not occurring through passive diffusion. Next, real‐time capture of the early interactions between EVs and each acceptor cell type was carried out at 37°C which showed an increasing emission intensity at the PM over time (Figure S3, SI Movies S1 and S2). Using phasor plot analysis of FLIM images at 30 min post‐incubation confirmed that only the disaggregated form of **1**, with a lifetime of 2.7 ns, was detected in the PM (Figure S4).

**FIGURE 10:**
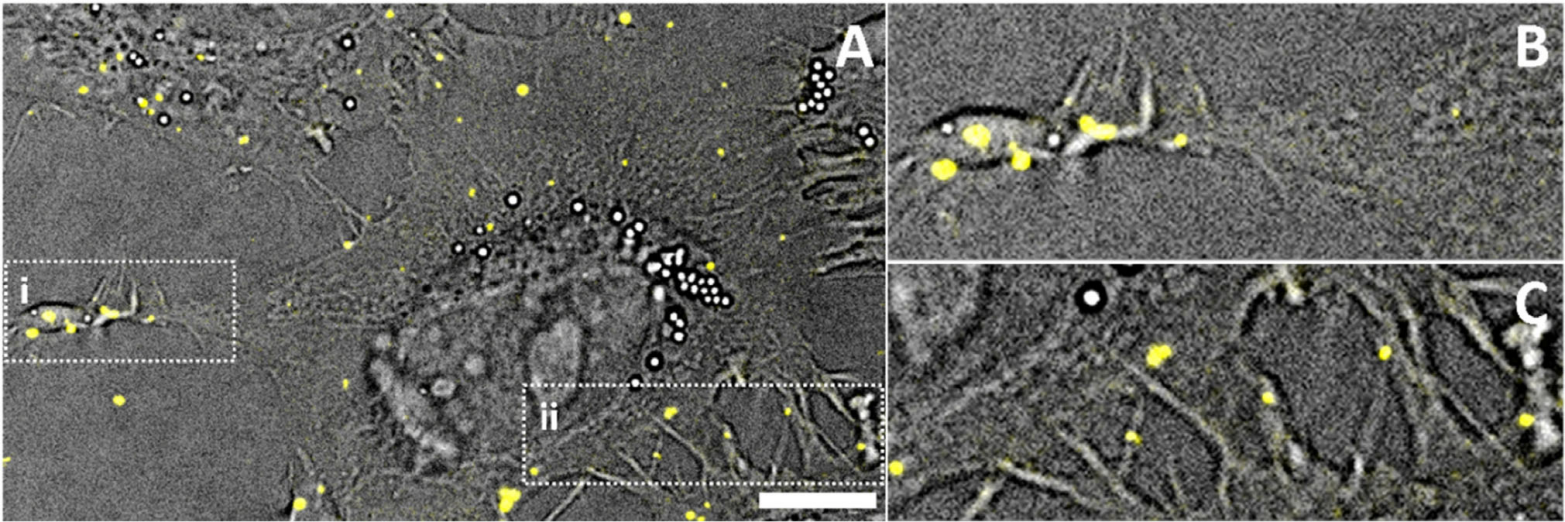
Observing first contact of endogenously loaded EVs (yellow colour) with the plasma membrane of acceptor HeLa cells at 10 min post‐treatment. (A) Confocal image (yellow) superimposed on brightfield image of HeLa cells. (B) Expansion view of boxed area (i) showing EVs in contact with cellular protrusion. (C) Expansion view of boxed area (ii) showing EVs in contact with individual filopodia. Scale bar 10 μm.

However, the interaction between EVs and the PM occurs rapidly at physiological temperatures, making it challenging to capture the initial stages of vesicle–cell contact and uptake. Due to the speed of events, revised experimental conditions were used in which cells were first cooled to 4°C prior to treatment with EVs, placed on the microscope stage and a FOV selected. Following EV addition, the cells were incubated at 4°C for 30 min. After incubation, they were transferred to the microscope chamber maintained at room temperature, where they gradually warmed to ambient conditions during imaging. Under these initially reduced temperature conditions, cellular uptake of EVs is minimised, allowing for observation of vesicle accumulation at the cell surface and transfer into the cell as the temperature rises. Using this experimental approach, an enhanced observation of vesicle accumulation at the outer PM of the cell surface was achievable (Figure 10, for additional experiments, see SI Movie S4, S5 and S6).

**FIGURE 11:**
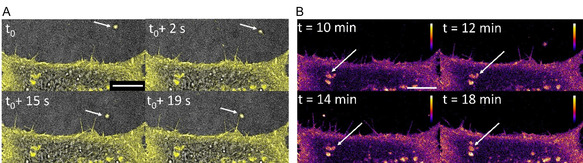
(A) Timelapse (commenced 10 min post‐treatment of cells with EVs) of superimposed confocal (yellow colour) on brightfield images showing first contact of EV (arrows) with an acceptor HeLa cell filopodia over 20 s (SI Movie S7). (B) Heat map of fluorescence intensity of a plasma membrane segment of an acceptor HeLa cell showing an increasing accumulation of **1** in cellular trafficking vesicles (arrows) (SI Movie S8). Scale bar 10 μm. For additional experiment examples, see SI Movies S9 and S10.

Commencing imaging at 4°C and allowing the temperature to gradually rise while imaging (72 frames per min), a longer acquisition series beginning 11 min after incubation with the labelled EVs was collected. Within this series, a 20 s segment is highlighted (Figure 11A, SI Movie S7), specifically capturing the interaction of a single EV with filopodia on the PM. Under these imaging conditions, EVs accumulation within internal vesicles below the inner leaf of the PM could be recorded over longer time periods. As shown in the timelapse fluorescence heat map of Figure 11B, an accumulation of **1** within internal trafficking vessels adjacent to the PM was notable, with a progressive increase in the fluorescence intensity observed below the PM and within these vesicles over time (SI Movie S8). This confirmed an efficient progression through the PM and delivery of the EV cargo to intracellular sites [66].

As control, following a 30 min incubation at 4°C with 0.5 µM fluorophore, a distinct difference in fluorescence patterns was observed (Figure S5). Under these exposure conditions, cells displayed relatively uniform intensity PM staining in comparison to labelled EVs which exhibited a more localised punctate staining of the cell surface.

**FIGURE 12:**
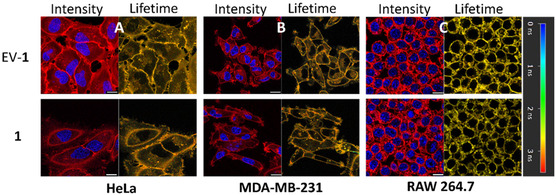
Confocal fluorescence intensity images of cells incubated with endogenously labelled EVs (red colour, top row) compared to cells incubated with 0.5 µM of **1** (red colour, bottom row) at 2 h post‐incubation with nuclei co‐stained with Hoechst (blue colour) and corresponding FLIM images (orange colour). (A) HeLa, (B) MDA‐MB‐231 and (C) RAW264.7 cells. Scale bars 10 µm. SeeFigures S6 and S7 for associated phasor plots for each fluorescence lifetime image and images taken at 2 and 5 h time points.

Next, imaging was conducted over a longer 5 h time scale post‐acceptor cell treatment with EVs to allow cross‐comparison between cell types. In all three cell lines, there was no discernible difference in intracellular fluorescence intensity distribution from the PM and internal vesicles at 30 min, 2 or 5 h time points (Figure 12, Figures S6 and S7). A co‐staining of nuclei with Hoechst 33 342 confirmed that no intranuclear emission from **1** was notable. Notably, when this time course was repeated using 0.5 μM **1** alone, i.e., not encapsulated within EVs, the intracellular distributions were the same as that of the EV delivered fluorophore. For completion, the lifetimes for all experiments were also recorded and were uniformly measured as 2.7 ns, irrespective of cell type or mode of delivery confirming that only the disaggregated form of **1** was being imaged (Figure 12, Figure S6). Taken together, these results show that while endogenously labelled EVs can effectively deliver the fluorophore cargo to the cell: irrespective of how the fluorophore is delivered, it is the cell that ultimately determines where the fluorophore gets localised on a subcellular level once internalised [67].

## 3 Conclusion

In this study, the conventional order of first isolation and then labelling for fluorescent EV production was reversed in favour of having cells self‐label their EVs with fluorophore **1** prior to releasing them into the surrounding media from which they could be isolated. A gained appreciation of the disaggregation and aggregation photophysical characteristics of **1**, which are controlled by concentration and the biochemical constitution of its surrounding environment, has been exploited to confirm the integrity of its endogenously labelled EVs. Importantly, the aggregates of **1** which have a distinct emission and lifetime (810 nm, 0.1 ns) could be detected in solution and cell experiments clearly distinguishing them from the non‐aggregate dissociated form (720 nm, 2.7 ns). When treating cells with a high 5 μm concentration of **1** (the first step for endogenously labelling), this dual‐lifetime behaviour allowed both forms to be simultaneously observed with the temporal changes tracked using phasor plot analysis. Following the broad intracellular distribution throughout donor cells, **1** was incorporated into EVs at the point of their biogenesis. By doing so, this facilitates an as natural as possible labelling of EVs allowing their isolation following efflux. After the ready‐labelled EV isolation and purification, a thorough characterisation of their emission wavelength and lifetime properties revealed that EV encapsulated **1** was in its molecular form and dispersed throughout the vesicles. More broadly, this established our microscopy approach to confirm that endogenously labelled EVs were not contaminated with fluorophore aggregates, which is a problematic issue with exogenous labelling. Photophysical characterisations of labelled EVs showed a single emission maximum and lifetime (720 nm, 2.7 ns), with co‐staining for membrane‐bound tetraspanin CD63 showing that **1** was localised in the lumen of the EVs, thereby confirming the integrity of our endogenous labelling method. This can provide a more accurate representation of EV uptake and cargo distribution in acceptor cell studies, circumventing the pitfalls of exogenous labelling by ensuring that EVs are never directly exposed to excess dye. Labelled EVs, from donor cells, were utilised in live‐cell microscopy experiments to image capture their contact with the PM of acceptor cells to their internalisation and accumulation within subcellular locales. Three different acceptor cell types, HeLa Kyoto, RAW264.7, and MDA‐MB‐231, were each in turn incubated with labelled EVs and timelapse imaged over 5 h using confocal microscopy. The first EV contact with filopodia and the PM of acceptor cells could be observed with increasing accumulation within and around filipodia. EV interaction with, and passage through, the PM could be recorded in temperature‐controlled experiments with an accumulation of **1** in trafficking cellular vesicles close to the inner leaf of the PM. Monitoring over 5 h showed a continual accumulation of **1** within the cell and that it was confined to cytoplasmic vesicles with no accumulation within the nucleus. It was discovered that there was no significant difference of internal distribution between cells treated with fluorophore alone and those treated with **1** encapsulated within EVs. These results indicate that the ultimate destination of the EV cargo is dependent on the cargo itself and on sorting processes inherent to cells. The positive outcomes from this work may serve as a foundation for further investigations into the molecular design requirements for endogenous loading of EVs with the encapsulation of bio‐responsive fluorophores, therapeutics and theranostic agents being future goals.

## 4 Material and Methods

### 4.1 General

Fluorophore **1** was synthesised following literature procedures (45). Cell culture media including DMEM, 1% L‐glutamine, PBS, fetal bovine serum (FBS), 1% penicillin‐streptomycin, 175 cm Easy Flasks F/T Angled and 75 cm Easy Flasks F/T Angled were purchased from Thermo Fisher Scientific, Dun Laoghaire, Dublin, Ireland. Human breast adenocarcinoma (MDA‐MB‐231; RRID:CVCL_0062), human cervical carcinoma (HeLa CCL‐2; RRID:CVCL_1922) and murine macrophage (RAW 264.7, TIB‐71; RRID:CVCL_0493) cell lines were obtained from ATCC. Sterile PES syringe filters (0.45 µm) were purchased from Fisher Scientific, Dublin 1, Ireland. Ultra‐centrifugal filter 100 kDa MWCO and 15 mL sample volume were purchased from Merck KGaA, Darmstadt, Germany. Absorbance spectra were recorded with a Varian Cary 50 scan ultraviolet–visible spectrometer. Emission/excitation spectra were recorded on a FluoroMax Plus spectrophotometer. Confocal and FLIM images were acquired on the Leica Stellaris 8 Falcon system (objective: Leica HC PL APO CS2 100X/1.40 oil immersion) Wetzlar, Germany. The white light laser was used to excite the fluorophores. Images were subsequently processed utilising the LASX FALCON (FLIM) software (version 4.8.1.29271).

### 4.2 Spectroscopic Analysis

Serial dilutions of polysorbate 20 (0%–1% w/v) were prepared in DMEM containing 5 µM of **1**. Fluorescence emission spectra were recorded for each solution, and the maximum fluorescence intensity at 720 nm was noted for each sample. Compound **1** was weighed and dissolved in MeOH, DMEM and 0.1% polysorbate 20 in DMEM of appropriate volume to produce stock solutions of 200 µM each. These stock solutions were further diluted with complete DMEM and supplemented with 10% FBS to a final concentration of 5 µM. Absorbance and fluorescence spectra were recorded (excitation 670 nm, slits excitation 5 nm and emission 5 nm). All scans were carried out at room temperature.

#### 4.2.1 Liposome Synthesis

Liposomes were synthesised by adding 1 mL of 0.22 µm filtered PBS (pH 7.4) to a commercially available lipid mixture (L‐α‐phosphatidylcholine, stearylamine and cholesterol at 7:2:1) at room temperature, followed by sonication for 5 min. The resulting cloudy suspension of liposomes were then treated with of compound **1** (5 μM) and subsequently dropped onto poly‐L‐lysine‐coated slides and imaged with an excitation at 684 nm to evaluate both the emission intensity, λ_max_ and the fluorescent lifetime of the stained liposomes. Imaging was performed using the Leica Stellaris 8 Falcon System (objective: Leica HC PL APO CS2 100X/1.40 oil immersion). For intensity steady‐state fluorescence intensity imaging, the laser power was maintained at 10% and two distinct spectral regions were inspected simultaneously from 700 to 750 nm, and 800–850 nm. For time‐resolved fluorescence imaging in FLIM, a laser power of 4% was used and the emission between 700 and 850 nm was obtained, with the stop condition set to five consecutive frames. Scanning speed remained at 400 Hz for all images. Emission spectra were recorded using excitation at 684 nm.

### 4.3 EV Cultivation and Isolation

MDA‐MB‐231 cells were seeded and cultured in a T‐175 flask until confluency, achieving a density of 2.2 × 10^7^ cells per flask. To obtain a single EV pellet, cells from three separate T‐175 flasks, each at confluency, were utilised. 5 µM of **1** in complete DMEM was used to incubate each T‐175 flask for 2 h. The cells in each flask were subsequently washed three times with pre‐warmed PBS, after which they were replenished with serum‐free DMEM and incubated for 48 hr to allow EV production. The conditioned medium containing EVs were pre‐filtered by passing the contents of each T‐175 flask through a 0.45 µm sterile filter to exclude debris and dead cells from the medium. The filtered medium was then collected in Ultra (AU) filter (100 kDa MWCO) tubes and enriched as follows: 2 mL of sterile PBS was added to the AU filter and centrifuged at 4000 × *g* for 10 min in a swinging bucket rotor. PBS was removed from the bottom of the filter device, and the filtrate was aspirated from the collection tube. A total of 15 mL of pre‐clarified sample was added to the AU filer and centrifuged at 4000 × *g* for 30 min. The contents of the collection tube were discarded, and the filter device was washed with 15 mL of pre‐warmed PBS, followed by gentle pipetting to ensure thorough mixing. The suspension was then centrifuged at 4,000 × *g* for 30 min. The concentrated sample obtained from each tube formed a single EV pellet.

### 4.4 Characterisation of EV Size Using NTA

The average diameter of the isolated EVs was measured through NTA using a Malvern NanoSight NS300. For measurement, 100 μL of the isolated EV sample was diluted in 900 μL of serum‐free cell culture medium to achieve optimal particle concentration for analysis.

### 4.5 Fluorescent Imaging of EVs

20 µL of isolated endogenously labelled EV samples were dropped on a glass slide, where the fluorescent EVs were observed using the Leica Stellaris 8 Falcon System (objective: Leica HC PL APO CS2 100X/1.40 oil immersion). For standard confocal imaging, excitation was performed at 684 nm with the laser power maintained at 10%, and emission was detected within the 700–750 nm range. FLIM was carried out using a reduced laser power of 5%, with emission collected from 700 to 850 nm. FLIM acquisition was terminated after the capture of 10 consecutive frames. A uniform scanning speed of 400 Hz was used for all images. Emission spectra were acquired under the same excitation conditions (684 nm). For co‐staining, 100 μL of isolated EVs were incubated with 10 μL of CD63 monoclonal antibody (MEM‐259) conjugated to Alexa Fluor 488 (Thermo Fisher Scientific, Dun Laoghaire, Dublin, Ireland) for 12 h. After incubation, co‐stained EV samples were washed three times with PBS to remove unbound antibody prior to imaging using the aforementioned parameters.

### 4.6 Measuring the Concentration of fluorophore 1 in EVs

A calibration curve was generated for fluorophore **1** to allow for the quantification of its concentration within a single batch of isolated EVs. The EVs were collected from conditioned media pooled from three T‐175 flasks, each containing 30 mL of serum‐free media, following a 48‐h incubation period during which MDA‐MB‐231 cells secreted endogenously labelled EVs. Serial dilutions of fluorophore **1** were prepared at concentrations of 5 μM, 2.5, 1.25, 0.625, 0.3125 and 0.15625 μM in serum‐free DMEM supplemented with 0.1% (w/v) polysorbate‐20. Emission spectra were acquired following excitation at 680 nm, and fluorescence intensity was recorded at the emission maximum of 720 nm. A linear regression was applied to the calibration data, and the resulting standard curve was used to extrapolate the fluorophore concentration present in the labelled EVs.

### 4.7 Cell Culture and Live Cell Confocal and FLIM Microscopy

MDA‐MB‐231 cells, HeLa Kyoto cells and RAW264.7 cells were cultured in complete DMEM containing 1% L‐glutamine, 1% penicillin–streptomycin and 10% FBS at 5% CO_2_ and 37°C. Cells were seeded at a density of 1 × 10^4^ cells per well on an eight‐well Ibidi chamber slide and allowed to proliferate for 48 h prior to imaging. The acquisition of confocal images was performed using the Leica Stellaris 8 Falcon System (objective: Leica HC PL APO CS2 100X/1.40 oil immersion) using 4% laser power for excitation. Images were processed through the LASX FALCON (FLIM) software (version 4.8.1.29271). The fluorophore was excited at 684 nm, and two distinct spectral regions were inspected simultaneously from 700 to 750 nm, and 800 to 850 nm. Employing FLIM, the stop condition for photon accumulation was set to five consecutive frames. The white light laser was set at 684 nm to excite **1** with minimum power applied to avoid detector saturation. In the case of FLIM, the emission between 700 and 850 nm was acquired. The scanning speed setting for all images acquired was 200 Hz. Emission scans for the fluorophore in its molecular and aggregate forms were individually produced with 10% laser power. The results were repeated in triplicate with the average results used. To control the environmental temperature and CO_2_ concentration, the microscope chamber set up with the onstage incubator and CO_2_ metre, set to 37°C and 5% respectively. Cell‐seeded slides were positioned on the stage, and an ideal FOV with a selection of cells was adopted. Cell media from the well was aspirated and immediately replaced with 300 µL of 0.5 µM of **1** in serum‐free media, or 5 µM of **1** in complete media, or isolated EVs in serum‐free media, and the imaging protocol was initiated. Images were sequentially acquired at 5 min, 30 min, 2 h or 5 h, depending on the experiment being conducted. EV experiments were conducted at 4°C to investigate temperature‐dependent cellular interactions where cells were incubated with 300 µL of 0.5 µM of **1** in serum‐free media or 300 µL of isolated EVs in serum‐free media and maintained at 4°C for 30 min prior to incubation using the above parameters at room temperature.

### 4.8 Analysis of Images

Raw TIFF images were processed using ImageJ (NIH, v1.54g). Prior to analysis, measurement parameters were selected by navigating to *Analyze > Set Measurements*, selecting ‘Area’, ‘Mean Gray Value’ and ‘Min & Max Gray Value.’ Individual cells were manually outlined using the freehand selection tool, ensuring exclusion of neighbouring cell membranes and inclusion of all associated labelled filopodia for the selected cell. Mean fluorescence intensity values were obtained via *Analyze > Measure*. For quantitative comparison, only those cells for which the minimum and maximum grey values were consistent across all analysed samples were included in the calculation of average and standard deviation.

## Supporting Information

Additional supporting information can be found online in the Supporting Information section. Supplementary Information available: For additional EV imaging experiment replicates and timelapse movies, see https://doi.org/10.1039/x0xx00000x. **Supporting** **Fig. S1**: Absorbance and emission spectra of **1**. **Supporting** **Fig. S2**: MDA MB 231 cells 48 h post‐incubation with serum‐free media after labelling with **1**. **Supporting** **Fig. S3**: Incubation of acceptor cells with labelled EVs at 37 °C showing filopodia (white arrows) and labelled EVs (white circles) imaged 20 mins post‐incubation. (A) RAW 264.7 cells, (B) HeLa cells, (C) MDA‐MB‐231 cell membrane interaction with EVs, (D) HeLa cell membrane interaction with EVs. **Supporting** **Fig. S4**: FLIM images of cells incubated with endogenously labelled EVs, 30 min post‐incubation (scale bar 10 μm). **Supporting** **Fig. S5**: Intensity images of cells incubated with 0.5 μM of 1 compared to cells incubated with endogenously labelled EVs, 30 min post‐incubation. (A) HeLa, (B) MDA‐MB‐231 and (C) RAW264.7 (scale bar 10 μm) cells. Top row: cells incubated with 0.5 μM of 1. Bottom row: cell incubated with EVs. **Supporting** **Fig. S6**: FLIM phasor plots at 2 h incubation of unlabelled acceptor cells with labelled EVs. **Supporting** **Fig. S7**: Flu. Intensity/FLIM (EV vs. Control) and corresponding FLIM phasor plots at 5 h of incubation of unlabelled acceptor cells with labelled EVs. **Supporting** **Movie S1**: Time lapse of confocal and brightfield microscopy showing increasing fluorescence intensity (yellow colour) at the plasma membrane of an acceptor MDA‐MB‐231 cell following internalisation of labelled EVs over 5 min. **Supporting** **Movie S2**: Time lapse of confocal and brightfield microscopy showing increasing fluorescence intensity (yellow colour) at the plasma membrane of an acceptor RAW 264.7 cells following internalisation of labelled EVs over 10 min. **Supporting** **Movie S3**: Time lapse of confocal and brightfield microscopy showing increasing fluorescence intensity (yellow colour) at the plasma membrane of an acceptor HeLa Kyoto cells following internalisation of labelled EVs over 15 min. **Supporting** **Movie S4**: Time lapse of confocal microscopy (yellow colour) of an acceptor HeLa Kyoto cell showing EV accumulation at the outer plasma membrane with gradual endocytosis of labelled EVs over 8 min. **Supporting** **Movie S5**: Time lapse of superimposed confocal (yellow colour) and brightfield microscopy of a portion of the plasma membrane of an MDA‐MB‐231 acceptor cell showing EV accumulation at the outer plasma membrane with gradual endocytosis of labelled EVs over 10 min. **Supporting** **Movie S6**: Time lapse of confocal (yellow colour) and brightfield microscopy of a portion of the plasma membrane of an MDA‐MB‐231 acceptor cell showing EV accumulation at the outer plasma membrane with gradual endocytosis of labelled EVs over 20 min. **Supporting** **Movie S7**: (movie of paper Figure 11A): Timelapse of superimposed confocal (yellow colour) on brightfield image showing first contact of EV with acceptor HeLa cell filopodia over 23 s. **Supporting** **Movie S8**: (movie of paper Figure 11B): Heat map of fluorescence intensity (lower intensity – purple colour; higher intensity – yellow/white colour) of a segment of the plasma membrane of an acceptor HeLa cell showing an increasing accumulation of 1 in cellular trafficking vesicles over 22 min. **Supporting** **Movie S9**: Time lapse of confocal (yellow colour) and brightfield microscopy of a portion of a labelled EV (circled) entering an acceptor cell. **Supporting** **Movie S10**: Time lapse of confocal (yellow colour) and brightfield microscopy of a portion of the plasma membrane of an acceptor cell showing EV accumulation at the outer plasma membrane with gradual endocytosis of labelled EVs over 20 min.

## Author Contributions

**Donal F. O’Shea**: conceptualisation, funding acquisition and supervision. **Eden Booth** and **Donal F. O’Shea**: writing original manuscript draft. **Eden Booth**: spectroscopic and cell imaging experiments, EV isolation and characterisation, image data analysis, and preparation of figures. **Massimiliano Garre**: imaging data analysis and microscopy training. **Dan Wu**: synthesis of compound **1**.

## Funding

This work was supported by Science Foundation Ireland (Grant 18/RI/5723), Irish Research Council and Disruptive Technology Innovation.

## Conflicts of Interest

DFOS has a financial interest in patents filed and granted relating to NIR‐fluorophores.

## Supporting information

Supplementary Material

## Data Availability

The data that support the findings of this study are available from the corresponding author upon reasonable request.
