# Supplementary material for: Endogenous Labelling of Extracellular Vesicles and Image Capture of Their Interactions With Acceptor Cells
Source: Chembiochem. 2026 Apr 4;27(7):e70292. doi: 10.1002/cbic.70292 (PMC13050282; doi:10.1002/cbic.70292)
Supplement: Supplementary file 1 — Supplementary Material [file CBIC-27-e70292-s001.zip › cbic.70292-Sup-0001-supdata-S1/DOShea SI_revised_2.pdf]

Endogenous Labelling of Extracellular Vesicles and Image Capture of their Interactions with Acceptor Cells

Eden Booth, Massimiliano Garre, Dan Wu, Donal F. O'Shea*

Department of Chemistry, RCSI, University of Medicine and Health Sciences, 123 St Stephen's Green, Dublin 2, Ireland.

Contents

Figure S1: Absorbance and *emission spectra of **1***

Figure S2: *MDA MB 231 cells 48 h post-incubation with serum-free media after labelling with **1***

Figure S3: *Incubation of acceptor cells with labelled EVs at 37 °C showing filopodia (white arrows) and labelled EVs (white circles) imaged 20 mins post-incubation. (A) RAW 264.7 cells, (B) HeLa cells, (C) MDA-MB-231 cell membrane interaction with EVs, (D) HeLa cell membrane interaction with EVs.*

Figure S4: *FLIM images of cells incubated with endogenously labelled EVs, 30 min post-incubation. (scale bar 10 µm)*

Figure S5: *Intensity images of cells incubated with 0.5 µM of 1 compared to cells incubated with endogenously labelled EVs, 30 min post-incubation. (A) HeLa, (B) MDA-MB-231, and (C) RAW264.7 (scale bar 10 µm) cells. Top row: cells incubated with 0.5 µM of **1**. Bottom row: cell incubated with EVs*

Figure S6: *FLIM phasor plots at 2 h incubation of unlabelled acceptor cells with labelled EVs.*

Figure S7: *Flu. Intensity/FLIM (EV vs. Control) and corresponding FLIM phasor plots at 5 h of incubation of unlabelled acceptor cells with labelled EVs.*

Movie Legends

Movie S1: Time lapse of confocal and brightfield microscopy showing increasing fluorescence intensity (yellow colour) at the plasma membrane of an acceptor MDA-MB-231 cell following internalisation of labelled EVs over 5 min.

Movie S2: Time lapse of confocal and brightfield microscopy showing increasing fluorescence intensity (yellow colour) at the plasma membrane of an acceptor RAW 264.7 cells following internalisation of labelled EVs over 10 min.

Movie S3: Time lapse of confocal and brightfield microscopy showing increasing fluorescence intensity (yellow colour) at the plasma membrane of an acceptor HeLa Kyoto cells following internalisation of labelled EVs over 15 min.

Movie S4: Time lapse of confocal microscopy (yellow colour) of an acceptor HeLa Kyoto cell showing EV accumulation at the outer plasma membrane with gradual endocytosis of labelled EVs over 8 min.

Movie S5: Time lapse of superimposed confocal (yellow colour) and brightfield microscopy of a portion of the plasma membrane of an MDA-MB-231 acceptor cell showing EV accumulation at the outer plasma membrane with gradual endocytosis of labelled EVs over 10 min.

Movie S6: Time lapse of confocal (yellow colour) and brightfield microscopy of a portion of the plasma membrane of an MDA-MB-231 acceptor cell showing EV accumulation at the outer plasma membrane with gradual endocytosis of labelled EVs over 20 min.

Movie S7: (movie of paper Figure 11A): Timelapse of superimposed confocal (yellow colour) on brightfield image showing first contact of EV with acceptor HeLa cell filopodia over 23 s.

Movie S8: (movie of paper Figure 11B): Heat map of fluorescence intensity (lower intensity - purple colour; higher intensity - yellow/white colour) of a segment of the plasma membrane of an acceptor HeLa cell showing an increasing accumulation of **1** in cellular trafficking vesicles over 22 min.

Movie S9: Time lapse of confocal (yellow colour) and brightfield microscopy of a portion of a labelled EV (circled) entering an acceptor cell.

Movie S10: Time lapse of confocal (yellow colour) and brightfield microscopy of a portion of the plasma membrane of an acceptor cell showing EV accumulation at the outer plasma membrane with gradual endocytosis of labelled EVs over 20 min.

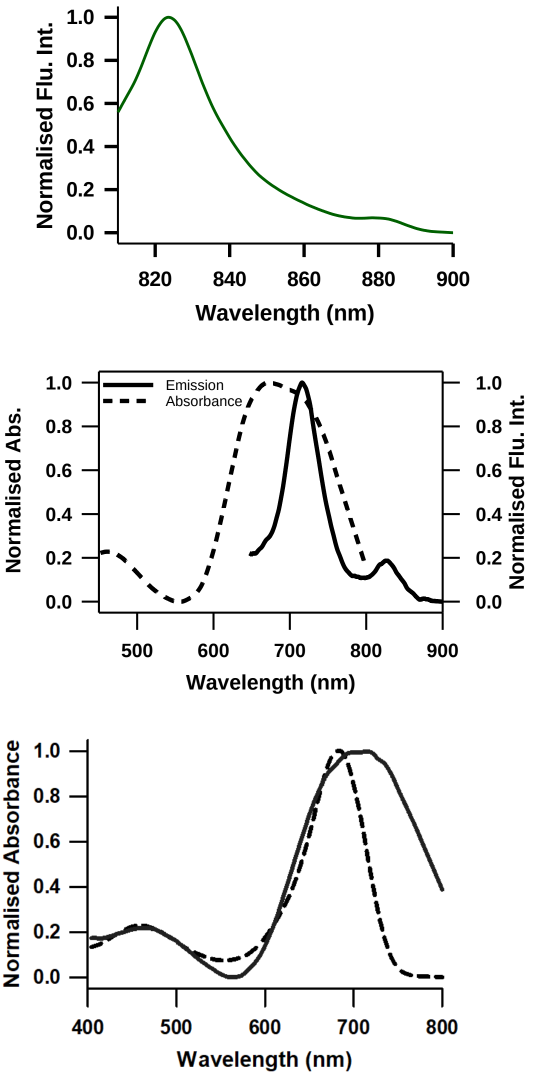

Figure S1. Top panel: Emission spectrum of 100 µM of **1** excited at 780 nm (10 nm slit width). Middle panel: Absorbance (black trace) and emission (red trace) spectra of **1** in PBS. Bottom panel: Comparison of absorbance spectra of **1** in MeOH (dashed trace) and PBS (solid trace).

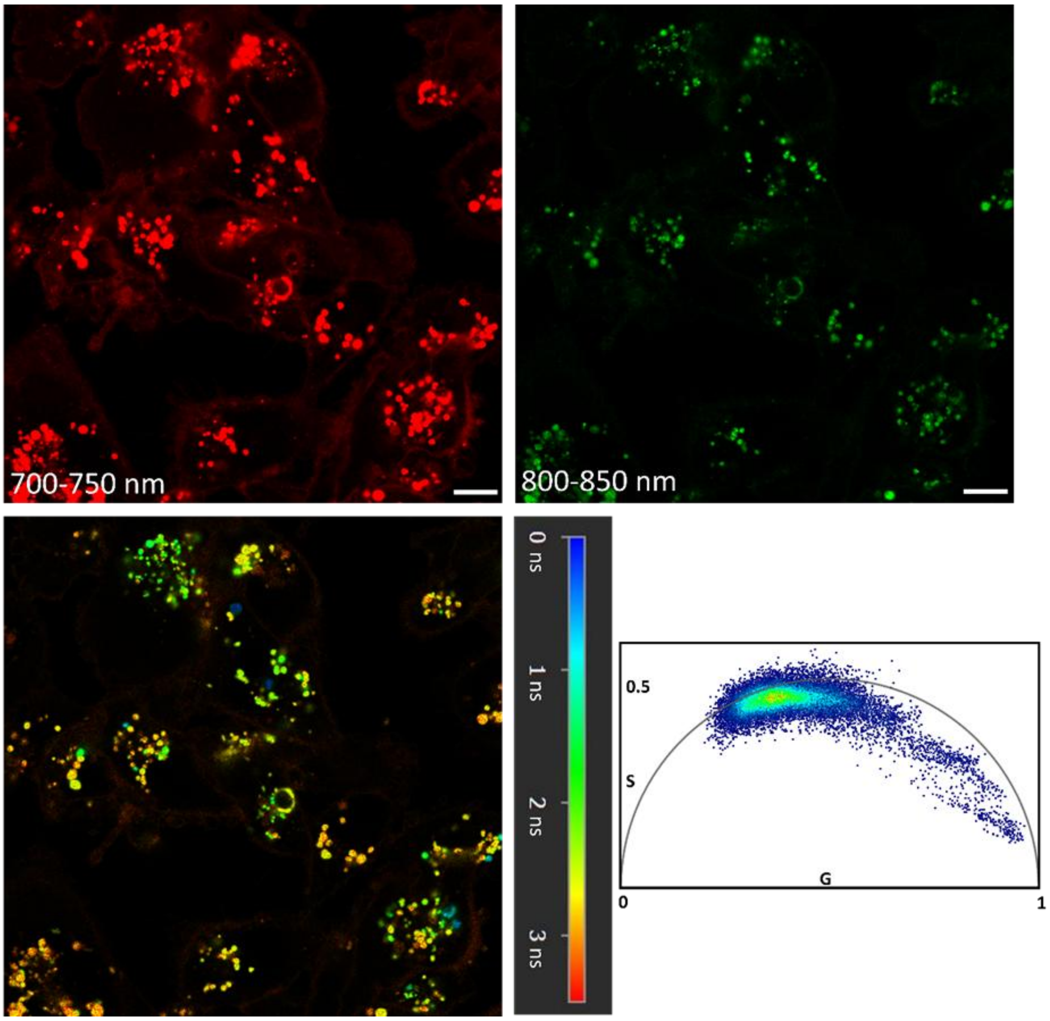

Figure S2. MDA MB 231 cells 48 h post-incubation with serum-free media after labelling with **1** (scale bar 10 µm).

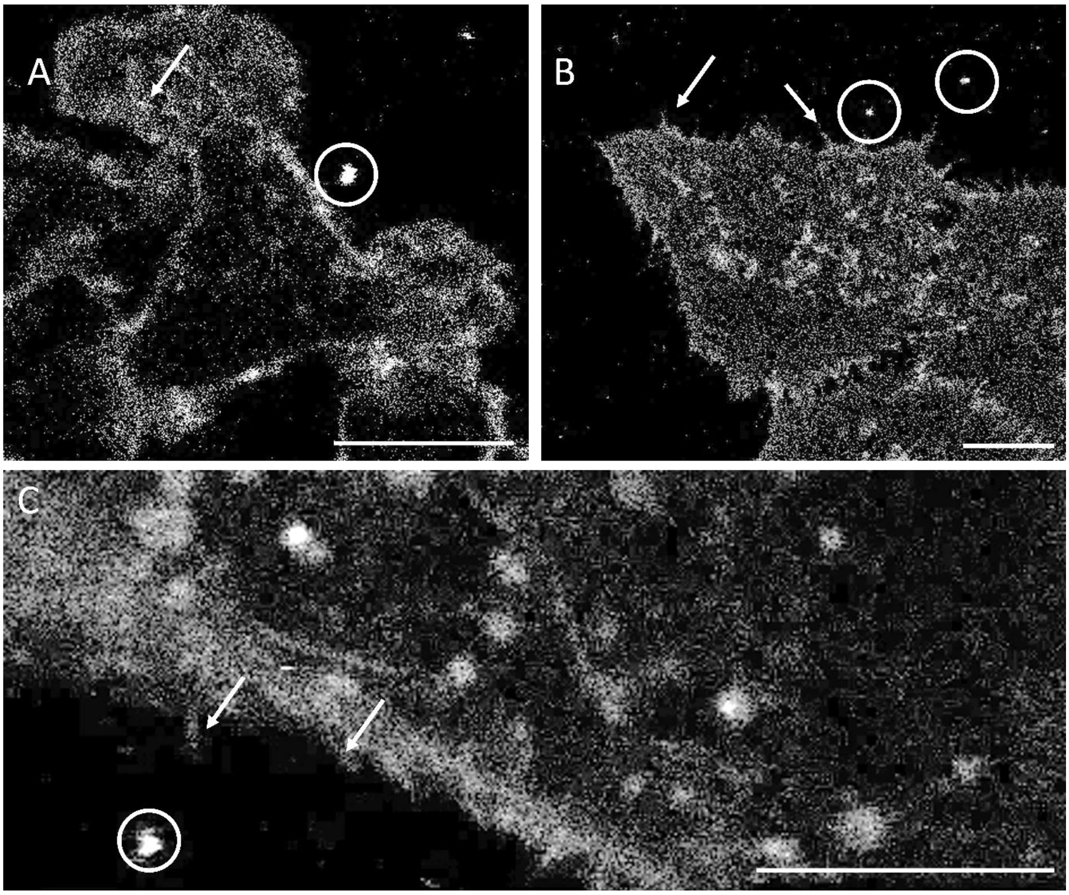

Figure S3. Incubation of acceptor cells with labelled EVs at 37 °C showing filopodia (white arrows) and labelled EVs (white circles) imaged 20 mins post-incubation. (A) RAW 264.7 cells (scale bar 5 µm), (B) HeLa cells (scale bar 10 µm), (C) MDA-MB-231 cell membrane interaction with EVs (scale bar 5 µm).

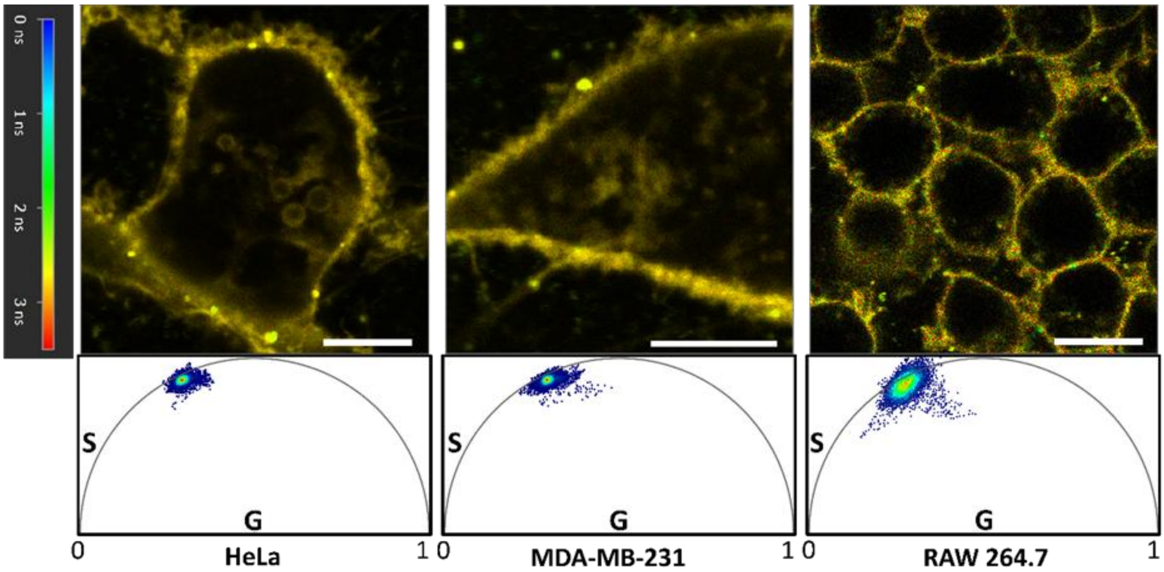

Figure S4: FLIM images of cells incubated with endogenously labelled EVs, 30 min post-incubation (scale bar 10 µm).

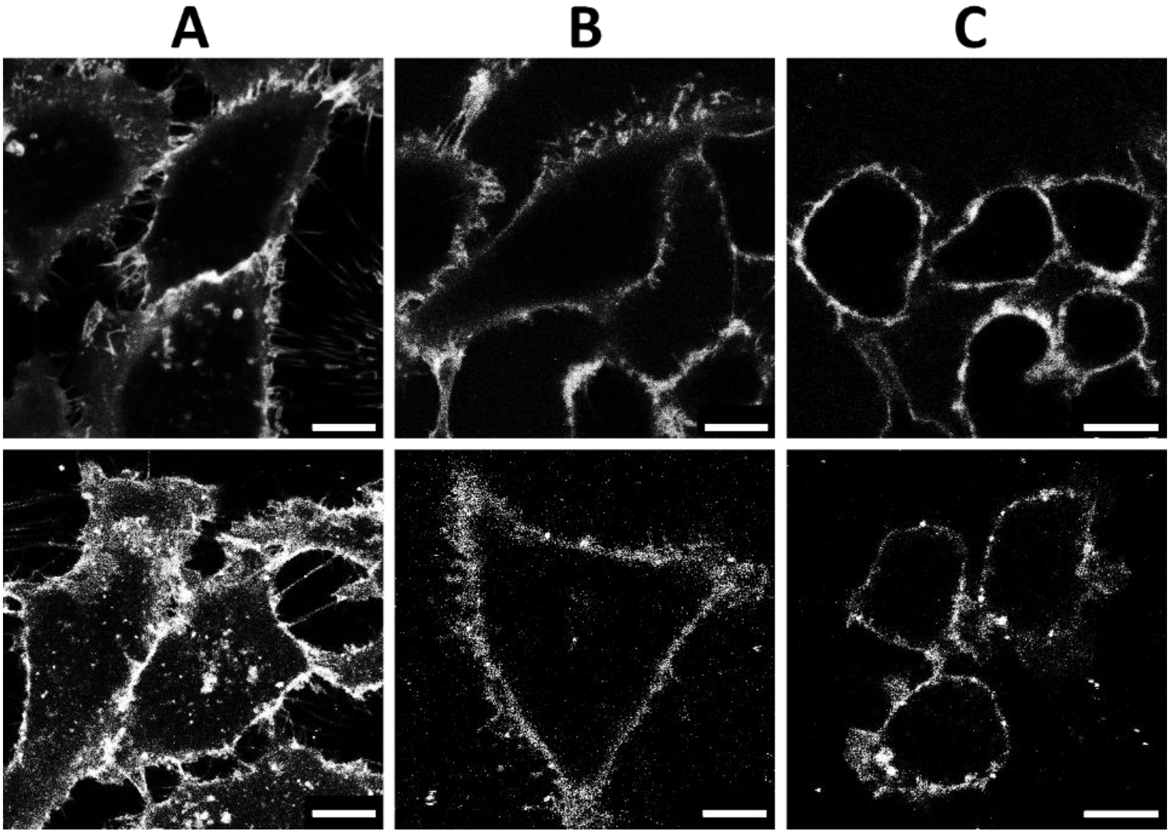

Figure S5: Intensity images of cells incubated with 0.5 µM of 1 compared to cells incubated with endogenously labelled EVs, 30 min post-incubation. (A) HeLa, (B) MDA-MB-231, and (C) RAW264.7 (scale bar 10 µm) cells. Top row: cells incubated with 0.5 µM of **1**. Bottom row: cell incubated with EVs.

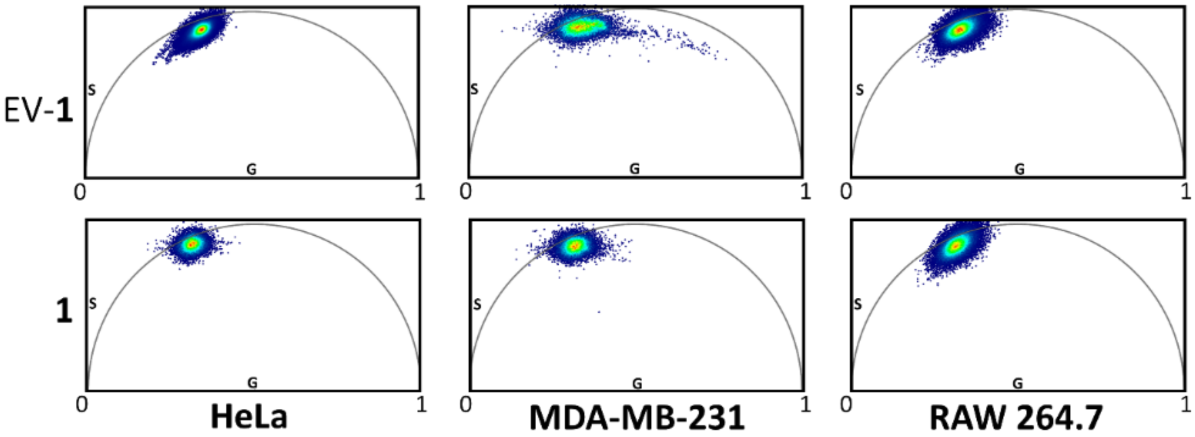

Figure S6: FLIM phasor plots at 2 h incubation of unlabelled acceptor cells with labelled EVs.

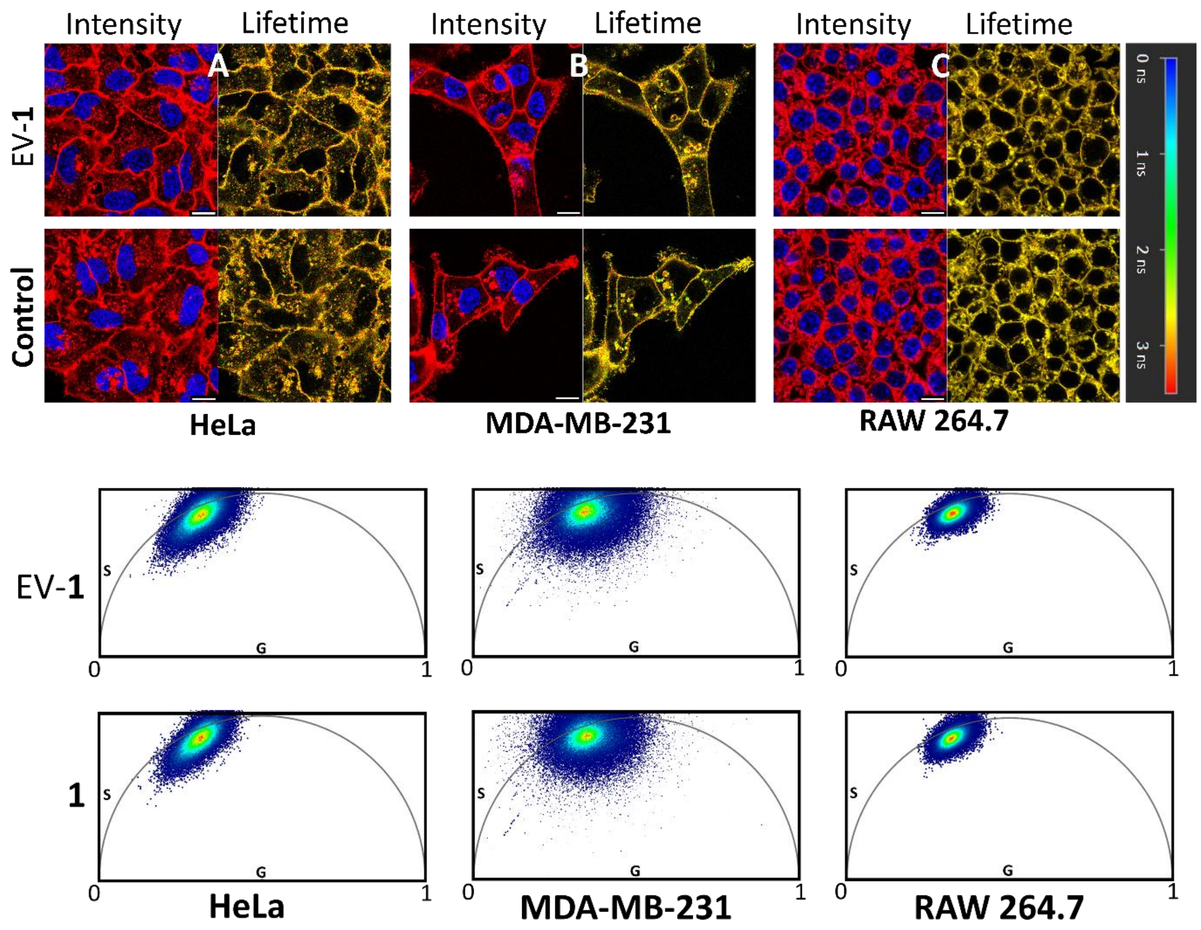

Figure S7: Flu. Intensity/FLIM (EV vs. Control) and corresponding FLIM phasor plots at 5 h of incubation of unlabelled acceptor cells with labelled EVs. Scale bars 10 µm.
